# Unbalanced Roles of Fungal Aggressiveness and Host Cultivars in the Establishment of the Fusarium Head Blight in Bread Wheat

**DOI:** 10.3389/fmicb.2019.02857

**Published:** 2019-12-11

**Authors:** Francis Fabre, Joerg Bormann, Serge Urbach, Sylvie Roche, Thierry Langin, Ludovic Bonhomme

**Affiliations:** ^1^Université Clermont Auvergne, INRAE, UMR 1095 Genetics, Diversity and Ecophysiology of Cereals, Clermont-Ferrand, France; ^2^Department of Molecular Phytopathology, Institute of Plant Science and Microbiology, University of Hamburg, Hamburg, Germany; ^3^Functional Proteomics Platform, Institute of Functional Genomics, CNRS UMR 5203 INSERM U661, Montpellier, France; ^4^INRAE, Unité Experimentale 1375, Phénotypage au Champs des Céréales (PHACC), Clermont-Ferrand, France

**Keywords:** *Fusarium graminearum*, *Gibberella zeae*, aggressiveness, proteomics, effector proteins, plant-pathogen interactions, bread wheat

## Abstract

Fusarium head blight (FHB), caused mainly by *Fusarium graminearum*, is the foremost destructive disease of cereals worldwide. Effector-like molecules produced by *F. graminearum* play key roles in the infection process and are assumed to be one of the essential components of the pathogen’s aggressiveness. However, their nature and role in the disease are still largely misunderstood. As a mean to provide relevant information about the molecular determinism of *F. graminearum* aggressiveness, we surveyed three *F. graminearum* strains on three wheat cultivars contrasted by their susceptibility to FHB. *F. graminearum* strains revealed large differences in aggressiveness which were mostly unchanged when facing hosts of contrasted susceptibility, suggesting that their behavior rely on intrinsic determinants. Surveying the fungal mass progress and the mycotoxin production rate in the spikes did not evidence any simple relationship with aggressiveness differences, while clues were found through a qualitative and quantitative characterization of the three strain proteomes established *in planta* especially with regards to early synthesized putative effectors. Independently of the wheat cultivar, the three *F. graminearum* strains produced systematically the same protein set during the infection but substantial differences in their abundance enabled the categorization of fungal aggressiveness. Overall, our findings show that the contrasts in *F. graminearum* aggressiveness were not based on the existence of strain-specific molecules but rather on the ability of the strain to ensure their sufficient accumulation. Protein abundance variance was mostly driven by the strain genetics and part was also influenced by the host cultivar but strain by cultivar interactions were marginally detected, depicting that strain-specific protein accumulations did not depend on the host cultivar. All these data provide new knowledge on fungal aggressiveness determinants and provide a resourceful repertoire of candidate effector proteins to guide further research.

## Introduction

Plants are constantly subjected to biological pressures that could compromise their development. Plant and pathogen interests are antagonist, thus generating an evolutionary dynamic between the two, promoted by the confrontation of the plant resistance with the ability of the pathogen to induce disease through an important variety of pathogenicity factors. Aggressiveness constitutes the quantitative component of pathogenicity and is defined as the degree of damage caused by the pathogen to the host (Van der Plank, 1968; Pariaud et al., 2009; Delmas et al., 2016). When a plant-pathogen interaction is established, proteins called effectors are synthesized to promote pathogenicity by suppressing the host’s immune response and inducing physiological manipulation of the plant (Kamoun, 2006). Effector proteins can be localized in the cell wall surface or secreted directly into the plant cells to target specific host functions (Kamoun, 2006; Lo Presti et al., 2015; Sperschneider et al., 2016; Jones et al., 2018). They can also play crucial roles in the degradation of complex compounds like the plant cell wall, in the initiation of infection and for obtaining nutrients from plant host reserves (Hao et al., 2019). Effector proteins are probably an essential component of the pathogen’s aggressiveness, nevertheless the molecular mechanisms underlying their functions are for the most part unknown, especially in fungus (Yi and Valent, 2013; van Schie and Takken, 2014; Jia and Tang, 2015; Lo Presti et al., 2015).

One relevant pathosystem for assessing the importance of pathogen’s aggressiveness during the interaction with the host is the relationship between bread wheat and *Fusarium graminearum* Schwabe (*Hypocreales*: *Nectriaceae*) (teleomorph: *Gibberella zeae*). *F. graminearum* is the most prominent causal agent of the FHB in Europe, Canada, and United States (McMullen et al., 1997; Brennan et al., 2003; Steiner et al., 2017). Severe outbreaks regularly result in significant yield losses (Parry et al., 1995; Xu and Nicholson, 2009; McMullen et al., 2012; Chen et al., 2019), as well as altering nutritional grain quality and inducing a major health problem throughout the food chain *via* grain contamination by mycotoxins (Liu et al., 2019). DON is the most commonly found toxin in cereals (Placinta et al., 1999). Previous works showed that DON could have a role in fungal spread beyond the initial infection (Bai et al., 2002) by facilitating the spreading of *F. graminearum* from spikelets into the rachis which might induce the switch from biotrophy to necrotrophy (Bönnighausen et al., 2018). DON is also known to allow the inhibition of host protein synthesis (Walter et al., 2010), and is believed to be an aggressiveness factor rather than a pathogenicity factor (Proctor et al., 1995; Pasquet et al., 2016).

Although *F. graminearum* strains are not all identical in their ability to induce disease (Carter et al., 2002; Goswami and Kistler, 2005), the molecular mechanisms and life traits that determine the fungal aggressiveness level are always very controversial according to the authors. Classically, *F. graminearum* variation in aggressiveness is measured with severity variables such as the percentage of spikelets infected or the size of the visual symptom (Cumagun et al., 2004; Saville et al., 2012). Mycotoxins production is also considered as a FHB aggressiveness component (Proctor et al., 1997, 2002; Mesterházy, 2002; Burlakoti et al., 2007; Shin et al., 2018). Molecular approaches have also been used to characterize differences in *F. graminearum* strain aggressiveness at the genome (Carter et al., 2002; Gale et al., 2002; Cumagun et al., 2004; Laurent et al., 2017, 2018) or at the transcriptome scale (Harris et al., 2016; Puri et al., 2016). Many other studies have also identified *F. graminearum* genes involved in pathogenicity and some appeared to have only a quantitative effect (i.e., aggressiveness-related genes) (Pariaud et al., 2009). These genes encode secreted proteins and effectors that may play roles in the infection course (Krijger et al., 2014; Lu and Edwards, 2015; Chetouhi et al., 2016; Fabre et al., 2019). Furthermore, previous genomics studies have identified more than 600 genes coding for secreted proteins (Brown et al., 2012; King et al., 2015). Some have been identified at the proteome level (Lowe et al., 2015; Fabre et al., 2019) suggesting that *F. graminearum* could synthesize a large number of proteinous effectors.

In a previous study, we investigated the molecular dialogue dynamics taking place during the early stages of the FHB progress in bread wheat (Fabre et al., 2019). This has highlighted dual protein regulations between 48 hpi and 72 hpi both in *F. graminearum* and in wheat, emphasizing that regulated *F. graminearum* proteins could dynamically adjust to the plant physiological responses (Fabre et al., 2019). *Fusarium graminearum* effectors have been shown to be accumulated at specific stages of infection to achieve precise roles in the progress of the interaction, especially at 72 hpi during symptoms appearance (Fabre et al., 2019). However, this previous study was carried out on only one aggressive strain and one susceptible wheat cultivar. Evaluating the specificity of these proteome adjustments in hosts and pathogens contrasting for their susceptibility and aggressiveness, respectively, represents a powerful lever to identify the molecular determinants that drive the development of the disease.

The aim of this work is to question the potential links between the intrinsic characteristics of different *F. graminearum* strain proteomes with their respective aggressiveness *in planta*, as well as the impact of different wheat genetic backgrounds on the accumulation of the pathogen proteins. The qualitative and quantitative characterizations of the three *F. graminearum* proteomes at 72 hpi were performed with a special interest on effectors and proteins coded by known *F. graminearum* aggressiveness-related genes. In addition, three variables classically used for the *F. graminearum* aggressiveness determination were described, (i) the severity of symptoms induced by the strains, (ii) the fungal mass development *in planta*, and, (iii) the DON synthesis rate of each strain. The joint analysis of all these data provide new insights into the determinism of *F. graminearum* aggressiveness at the early stage of the disease progress.

## Materials and Methods

### Plant Growth and *Fusarium graminearum* Inoculation

Experiments were conducted on three wheat cultivars of contrasting susceptibility to FHB, including in decreasing order of susceptibility cv. Recital, cv. Cadenza and cv. Renan as ranked from previous field observations. Recital and Renan are among the most contrasted cultivars of the french wheat collections (Gervais et al., 2003) while Cadenza is considered as intermediate. For each wheat cultivar, seeds were sown in buckets and kept at 20°C to allow germination. Vernalization was performed at 4°C for 8 weeks, then plantlets were transplanted in 4-L pots and transferred in a growth cabinet with optimal conditions to allow tillering and synchronized flowering. Twenty four plants per wheat cultivar were prepared for a total of 72 wheat plants divided in three randomized complete blocks in the growth cabinet and surrounded by additional plants to control any edge effects. Automatic watering was installed, and the daily photoperiod was set at 16-h daylight for a temperature of 20°C and 8-h darkness at 18°C. Relative humidity was maintain at 80% during day and night.

*Fusarium graminearum* strains MDC_Fg1, MDC_Fg13, and MDC_FgU1, all originating from France, were selected for their contrasting aggressiveness (based on field observations). The preparation of the *F. graminearum* inocula was performed as described in Fabre et al. (2019). The three strains were individually inoculated in six plants of each wheat cultivar i.e., six plants × 3 *F. graminearum* strains for a total of 18 plants per cultivar. For each cultivar, inoculation was performed at the mid-anthesis stage by depositing 10 μl of inoculum in the floral cavity of six contiguous spikelets located in the middle zone of three synchronized spikes per plant. The last six plants were inoculated with water and were used as control. For each cultivar×strain combination, the point-inoculated spikelets of the three spikes of three independent plants were specifically collected 72 h after the inoculation (hpi), while the ones of the three remaining plants were collected at 168 hpi. For each cultivar×strain combination, three biological replicates were designed at both time points; they corresponded to one individual plant which was characterized by the pool of all inoculated spikelets from three spikes. Fungal mass quantification in wheat spikes, *F. graminearum* DON synthesis rate and proteomics were performed on the same plant material.

### Symptom and Fungal Development Monitoring

**FIGURE 1:**
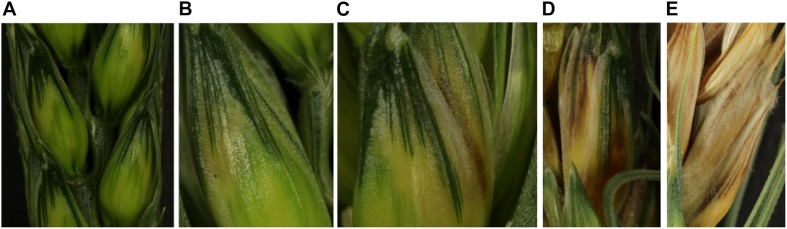
Determination of the symptom severity scale. **(A)** The picture represents spikelets with type-0 symptoms corresponding to asymptomatic spikelets. **(B)** Representation of type-1 symptoms on spikelet with the first yellowing spot. **(C)** The picture illustrates the type-2 symptoms characterized by a spikelet harboring the first browning spot. **(D)** The picture illustrates the type-3 symptoms characterized by a fully burnished spikelet. **(E)** The picture illustrates the type-4 symptoms characterized by a total drying of the spikelet with the visible mycelium outside the plant organ.

Symptoms were monitored by visual inspection and scored every 24 h from 0 to 168 hpi (hours post-inoculation) using a 5-level rating scale (Figure 1). A score of 0 was given to spikelets which had no visible symptom, a score of 1 was given when the first yellowing spot appeared, a score of 2 when a brown spot was visible, a score of 3 when browning gained the whole spikelet and a score of 4 when aerial mycelium became visible. Until 72 hpi, symptom monitoring was profiled on the whole set of plants (six plants for each cultivar×strain combination) while the range 96 hpi to 168 hpi was monitored from the remaining unsampled plants (three plants for each cultivar×strain combination).

Fungal mass was assessed at 72 and 168 hpi from the three biological replicates of each cultivar×strain combination, using qPCR method. For each biological replicate, the measurements made for each sample were performed nine times and were considered as technical replicates. Water-inoculated spikes were used as control. Frozen spikes from the same plant were mixed and finely ground under liquid nitrogen using pestle and mortar. The DNA-isolation was performed on 100 mg of ground material (i.e., 72 and 168 hpi) using CTAB buffer (100 mM Tris–HCl pH 8.0, 1.4 M NaCl, 20 mM EDTA and 2% cetyltrimethyl ammonium bromide). Samples were incubated at 65°C during 1 h and centrifuged at 10 000 *g* for 10 minutes at room temperature. DNA was recovered by adding 1 volume of chloroform and centrifuged as described above. Precipitation of the nucleic acids was performed using 1 volume of isopropanol and a centrifugation at 12 000 *g* for 20 minutes at 4°C. DNA has been cleaned with 70% ethanol and dissolved in 100 μL TE buffer (10 mM Tris HCl pH 8.0, 1 mM EDTA). The DNA concentrations of each sample were measured with the Tecan’s high performance Safire2^TM^ microplate reader. Concentrations of each sample were normalized by dilution to 100 ng/μL, 1 μl was used for qPCR using Roche LightCycler^®^ 480 SYBR Green I Master for each samples. Amplification was performed on the *F. graminearum* β-tubulin gene (Forward: TGCTGTTCTGGTCGATCTTG/Reverse: GACGGAAGTTTGGACGTTG) as described in Nguyen et al. (2013). Fungal DNA mass was estimated using a *F. graminearum* DNA dilution range (0.1–100 ng).

### DON Synthesis Rate by the Three *F. graminearum* Strains

DON synthesis rate per fungal mass unit was measured following the methodology described by Nguyen et al. (2013). DON measurement was realized at 72 and 168 hpi from the three biological replicates of each cultivar×strain combination and using water-inoculated samples as control. Each measurement was repeated nine times as technical replicates. For each sample, 50 mg of fresh ground material was suspended in 500 μl of distilled water and the extract was vortexed and centrifuged. A volume of 50 μl of the supernatant was used for DON quantification using the DON ELISA technique (RIDAscreen DON kits; R-Biopharm AG, Darmstadt, Germany. Detection range: 0.2 ppm to 6 ppm) and following the manufacturer’s instructions. In order to estimate the DON synthesis rate by fungal mass unit, each DON quantity determined by the ELISA method was subsequently normalized with the mass of mycelium quantified in the sample. Milligrams of mycelium per gram of fresh ground sample were computed by referring to the number of β-tubulin gene copies per milligram mycelium of the *F. graminearum* DNA dilution range (0.1–100 ng) as described in Voigt et al. (2007).

### Protein Extraction and LC-MS/MS Procedures

Denaturing protein extraction was achieved from the three biological replicates of each cultivar×strain combination collected at 72 hpi using the TCA/acetone procedure as described in Bonhomme et al. (2012). Protein solubilization was performed in an urea-thiourea buffer [6 M urea; 2 M thiourea; 100 mM ammonium bicarbonate; 1% Halt^TM^ Protease Inhibitor Cocktail 100X (Thermo Fisher Scientific 78429); 0.1% ProteaseMAX^TM^ Surfactant (Promega V2071)] by following the ratio 10 μL per mg of dry matter. Protein digestion were performed as described in Fabre et al. (2019). Tandem mass spectrometry analyses were achieved using a nanoESI Q Exactive^TM^ HF-X Hybrid Quadrupole-Orbitrap^TM^ Mass Spectrometer (Thermo Fisher Scientific 0726042) coupled with an Ultimate 3000 HPLC (Thermo Fisher Scientific). HPLC gradients and data acquisition parameters were set as described in Fabre et al. (2019).

### Identification and Quantification of Peptides and Proteins From MS/MS Data

Database searches were performed using X!Tandem^1^; 2010.01.01.4) specifying one possible miscleavage. Cys carboxyamidomethylation and Met oxidation were set as static and variable modification, respectively. Precursor mass and fragment mass tolerance were 10 ppm and 0.5 Da, respectively. Identifications were performed using a concatenated file including a contaminant database (trypsin, keratins, etc.), the MDC_Fg1 (13166 entries, 01/2019), MDC_Fg13 (13297 entries, 01/2019), and MDC_FgU1 (13014 entries, 01/2019) databases obtained from an in-lab re-sequencing of each *F. graminearum* strain (Alouane et al., 2018). To prevent peptides derived from plant proteins from being assigned to fungal proteins, a wheat database (IWGSC, v1.0 269472 entries, 04/2017) was also used for the identification. Identified proteins were parsed and grouped using the X!TandemPipeline v0.2.35 c++ (Langella et al., 2017). Data filtering was achieved according to a peptide *E*-value < 0.05. Proteins were reported when they displayed at least two different peptides in the same sample and when the protein *E*-value < 0.0001. The FDR (False Discovery Rate) at the peptide level assessed from searches against reversed amino acid sequences for each protein was smaller than 0.8 × 10−6. Protein and gene ontology annotations of the identified *F. graminearum* proteins were performed following the same methodology described in Fabre et al. (2019). Secretion features, subcellular localization predictions and functional annotations of identified proteins were conducted as described in Fabre et al. (2019). Relative quantification of peptides was achieved using the MassChroQ software (Valot et al., 2011) by extracting ion chromatograms as described in Bonhomme et al. (2012). The normalization was performed by dividing ratios by the total peptide abundance value in each LC-MS/MS run. Subsequent statistical analyses were performed on log10-transformed normalized data.

### Statistical Analyses

Results were analyzed with the R programing language v3.4.4 (R Core Team, 2018). For each time point and after validating the absence of any block/replicate effect, symptom severity monitoring were performed using generalized linear model following Poisson distribution:

log(Y)i⁢j⁢k=μ+S+iCv+j(S×iCv)j+ε,i⁢j⁢k

while fungal mass, DON synthesis rate and abundance variations of *F. graminearum* proteins were traced using two-way ANOVA with following linear model, after checking the absence of any block/replicate effect:

Y=i⁢j⁢kμ+S+iCv+j(S×iCv)j+ε,i⁢j⁢k

where *Y*_*ijk*_ refers to individual values, μ is the general mean of the variable considered, *S*_*i*_ is the effect of the *F. graminearum* strain inoculated, *Cv*_*j*_ is the effect of the wheat cultivar infected, *S*_*i*_×*Cv*_*j*_ is the interaction of the strain effect by the Cv, and ε*_*ijk*_* is the residual.

For each individual *F. graminearum* protein, *p*-values obtained from each effect were adjusted to control the FDR for independent test statistics (Benjamini and Hochberg, 1995). The FDR was < 0.05 corresponding, respectively, to *p*-values < 0.022, < 0.017, and < 0.00005 in the *S*, *Cv* and *S*×*Cv* effects, respectively. Fuzzy C-means clustering (Kumar and Futschik, 2007) of *F. graminearum* proteins showing significant abundance changes according to each effect tested (i.e., *S* and *Cv*) and their addition (i.e*., S*+*Cv*) was performed from Z-score transformed values and a fuzzification parameter of 2.

## Results

### *Fusarium graminearum* Strains Characterization Through Their Ability to Induce Symptoms, to Produce DON and Their Development *in planta*

**FIGURE 2:**
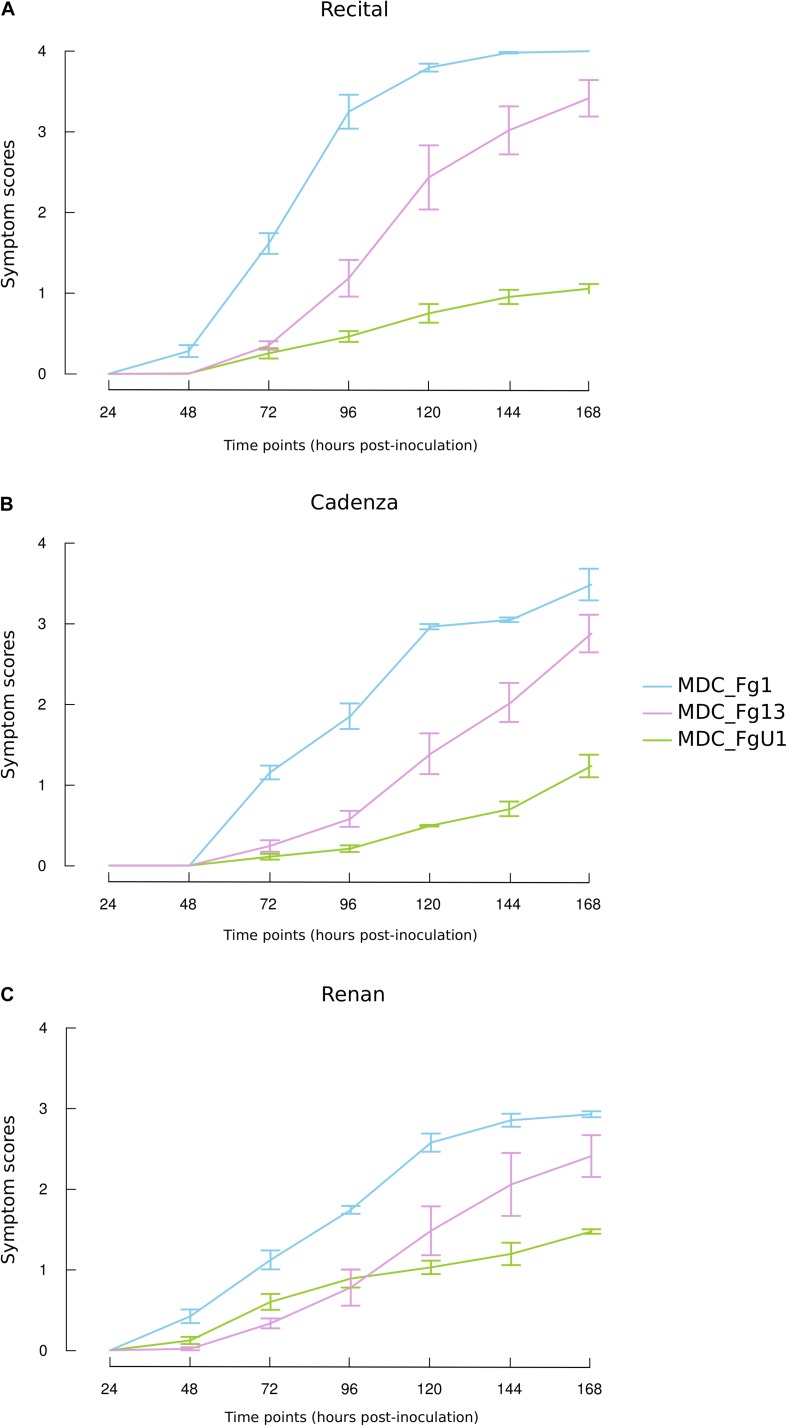
Symptom dynamics induced by the three *F. graminearum* strains on the three studied wheat cultivars **(A)** Recital, **(B)** Cadenza, and **(C)** Renan. Line plots show the course of the symptom severity observed for MDC_Fg1 (blue lines), MDC_Fg13 (pink lines), and MDC_FgU1 (green lines) on the three wheat cultivars. Values are means of six biological replicates until 72 hpi and means of three biological replicates from 96 hpi to 168 hpi. Each biological replicate was characterized from three spikes. Error bars indicate the confidence interval at 5% calculated for each time point independently.

**FIGURE 3:**
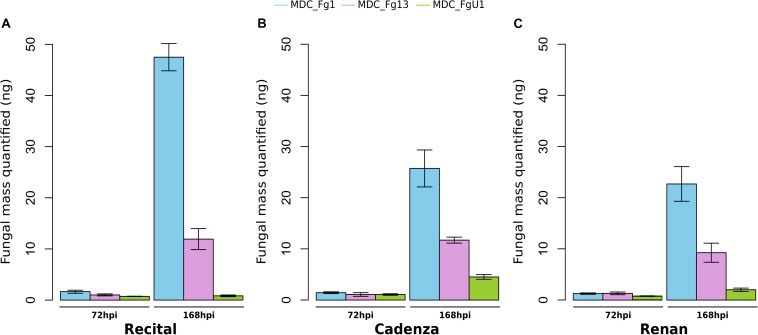
*Fusarium graminearum* strains development in infected wheat spikes. Fungal mass was assessed at 72 and 168 hpi in the cultivars Recital **(A)**, Cadenza **(B)** and Renan **(C)**, from the absolute quantification of the *F. graminearum* β-tubulin gene using qPCR (Nguyen et al., 2013). Values were estimated with a standard curve of 10-fold dilutions (ranging from 0.01–100 ng) of *F. graminearum* DNA purification for MDC_Fg1 (blue bars), MDC_Fg13 (pink bars) and MDC_FgU1 (green bars). Values are means of nine biological replicates computed for three biological replicates at 72 hpi and at 168 hpi. Each biological replicate was characterized from three spikes. Error bars indicate the confidence interval at 5% calculated for each time point independently.

**FIGURE 4:**
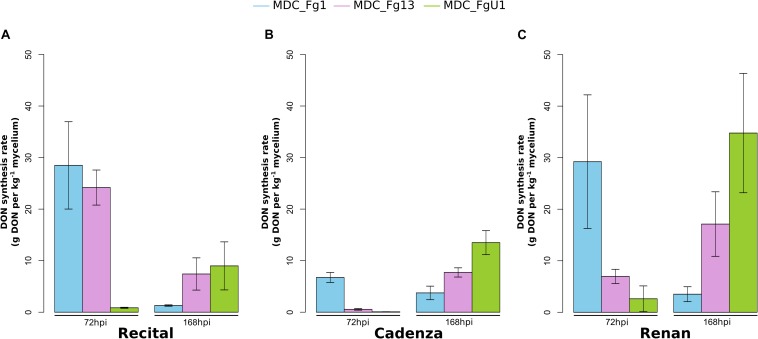
Fungal DON synthesis rate in infected wheat spikes. Bar graphs represent DON synthesis rate (in g of DON per kg^–1^ of fungal mycelium) in wheat heads infected with MDC_Fg1 (blue bars), MDC_Fg13 (pink bars) and MDC_FgU1 (green bars) in the cultivars Recital **(A)**, Cadenza **(B)**, and Renan **(C)**. Measurements were performed by enzyme-linked immunosorbent assay at 72 and 168 hpi, and normalized by the fungal mass quantified by qPCR. Values are means of nine biological replicates computed for three biological replicates at 72 hpi and at 168 hpi. Each biological replicate was characterized from three spikes. Error bars indicate the confidence interval at 5% calculated for each time point independently.

In this work, the evaluation of the aggressiveness of three *F. graminearum* strains were at first measured *via* three parameters: (i) the induction of symptoms, (ii) the development of the fungus in spike tissues, and (iii) the DON synthesis rate. Overall, these three parameters distinguished the three strains. MDC_Fg1 strain induced systematically the most intense symptoms, MDC_Fg13 strain induced intermediate ones while MDC_FgU1 strain produced the weakest ones (Figure 2). The earliest significant difference between strains were observed at 72 hpi and concerned the symptom appearance (Supplementary Table S1). At this time, wheat samples inoculated with MDC_Fg1 strain showed more severe symptoms than MDC_Fg13 and MDC_FgU1 with an average score of 1.33, 0.32, and 0.31, respectively, while fungal mass monitoring by qPCR did not identify any significant difference between the three strains (Figure 3). Concerning DON synthesis rate, MDC_Fg1 strain appeared to be the highest producer at 72 hpi with an average of 21.48 g per kg^–1^ of mycelium, 11.81 and 1.18 g.kg^–1^ for MDC_Fg13 and MDC_FgU1, respectively (Figure 4). At the end of the experimentation, symptom severity was significantly different between the three strains with an average score of 3.44, 2.88, and 1.18 that were monitored for MDC_Fg1, MDC_Fg13 and MDC_FgU1, respectively (Figure 2). At this stage, fungal mass monitoring showed different mean fungal development with an average of 31.91, 10.96, and 2.45 ng of mycelium for MDC_Fg1, MDC_Fg13 and MDC_FgU1, respectively. DON synthesis rate was also significantly different with 2.85, 10.74, and 17.12 g.kg^–1^ for MDC_Fg1, MDC_Fg13 and MDC_FgU1, respectively (Figure 4).

### *In planta* Characterization of the Three *F. graminearum* Strain Proteomes

**FIGURE 5:**
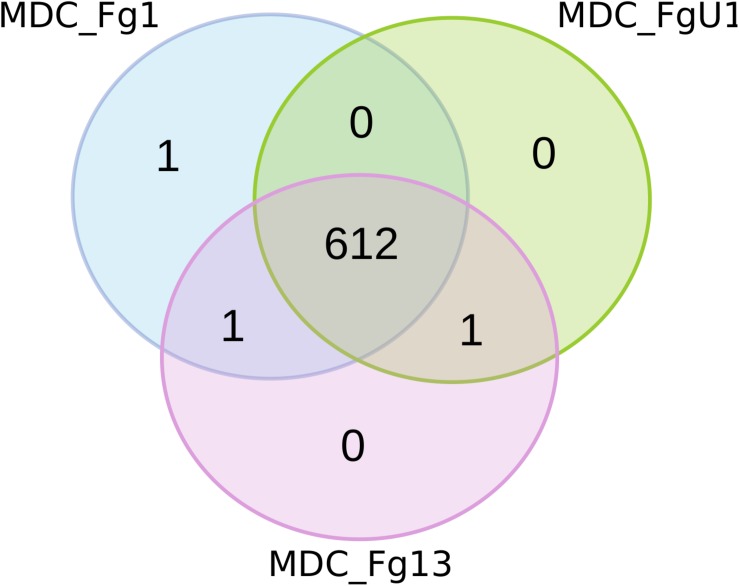
Number of fungal proteins identified for the three *F. graminearum* strains. For each quantified proteins, the Venn diagram represents the proteins that were strain-specific in the outer part of the circles and those quantified in samples inoculated with at least two different strains in the shared regions of the circles. MDC_Fg1, MDC_Fg13, and MDC_FgU1 proteomes are represented in blue, pink, and green color, respectively.

Proteomics analyses were performed at 72 hpi on the three wheat cultivars samples (i.e., Recital, Cadenza and Renan) inoculated with the three *F. graminearum* strains thus forming 9 *F. graminearum* strain×wheat cultivar combinations. Considering all *F. graminearum*-inoculated wheat samples, this study identified 615 unique *F. graminearum* proteins. Among all the identified proteins, 612 were shared by the three strains (Figure 5), two were identified in two different strains and only one was strain specific: FG001_00345, predicted to be a thioredoxin. Noteworthy, this protein has been identified in very low abundance in only two biological replicates of Recital samples inoculated with strain MDC_FG1.

**FIGURE 6:**
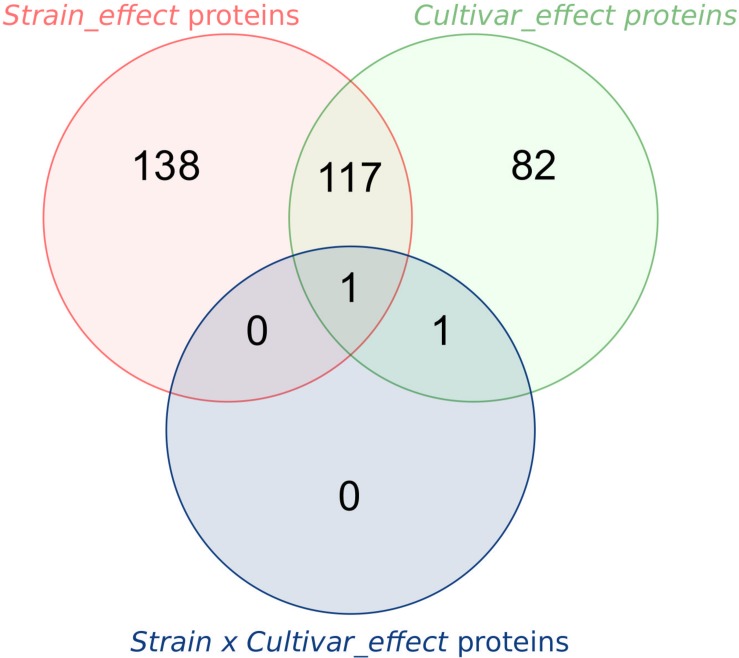
Number of *F. graminearum* proteins significantly impacted by the different effects in the two-way ANOVA. The Venn diagram shows the number of *F. graminearum* proteins whose abundance change was significant for each factor of the two-way ANOVA (*Strain_effect* proteins: FDR < 5%, *P*-value < 0.022; *Cultivar_effect* proteins: FDR < 5%, *P*-value < 0.017; and *Strain×Cultivar_effect* proteins: FDR < 5%, *P*-value < 0.00005).

Previous work has shown co-variations of fungal and wheat protein abundances between 48 and 72 hpi, suggesting that *F. graminearum* proteins and especially effectors, could participate in molecular mechanisms determining the fate of the interaction (Fabre et al., 2019). In order to better understand the specificity of these molecular mechanisms, here we performed a proteomics analysis at the end of this turning point (i.e., 72 hpi) to identify the proteins produced by three *F. graminearum* strains contrasted by their aggressiveness with a particular interest for predicted *F. graminearum* effectors. In all infected-wheat proteomics data aggregated, a total of 493 fungal proteins (corresponding to nearly 80% of the total identified proteins) were already identified in our previous work (Fabre et al., 2019). Similarly, 61 of the 72 predicted effectors identified during this work are common to both experiments. Regarding protein quantification, 340 (≈55% of all identified proteins) displayed significant abundance differences between *F. graminearum* strains. Two-way ANOVA were computed to evaluate the contribution of the “Strain” and “Cultivar” factors on the *F. graminearum* protein abundances. Abundances that were deemed significant to each factor enabled a categorization of the proteins according to the factor(s) that drive(s) the abundance differences. This included: (i) *F. graminearum* proteins whose abundance differences were specifically explained by the genetic background of the strain (Strain factor, *Strain_effect* proteins), (ii) *F. graminearum* proteins whose abundance differences were only explained by the host plant (Cultivar factor, *Cultivar_effect* proteins), (iii) *F. graminearum* proteins whose abundance differences were explained by both factors (*Strain*+*Cultivar*_*effect* proteins), and (iv) *F. graminearum* proteins whose abundance differences observed between strains were dependent on the host cultivar (*Strain*×*Cultivar_effect* proteins). Considering each factor individually, a total of 138 *Strain_effect* proteins, 82 *Cultivar_effect* proteins and 117 *Strain*+*Cultivar*_*effect* proteins and 2 *Strain*×*Cultivar*_*effect* proteins were identified (Figure 6 and Supplementary Table S2).

### How Does Fungal Genetics Shape the Protein Abundance Patterns?

**FIGURE 7:**
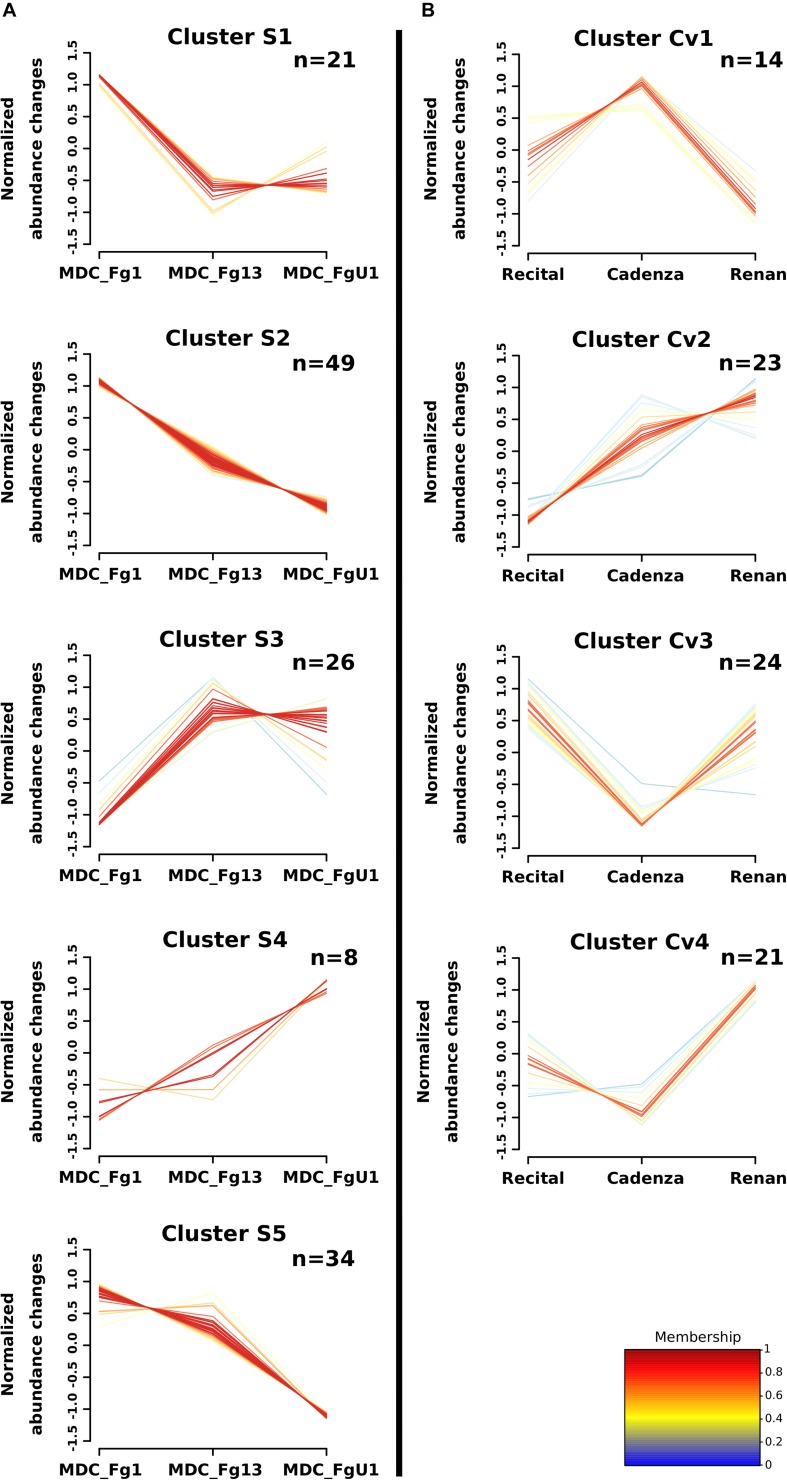
Clustering of *F. graminearum Strain_effect* and *Cultivar_effect* protein abundance patterns. Clustering was computed using the fuzzy C-means methods using *Z*-score transformed values to identify homogeneous patterns of protein abundance changes in **(A)** the *Strain_effect* proteins and in **(B)** the *Cultivar_effect* proteins. The number of proteins included in each cluster are specified in the right upper corner (n). The cluster membership of each protein profile is indicated by a color code from blue to red.

**FIGURE 8:**
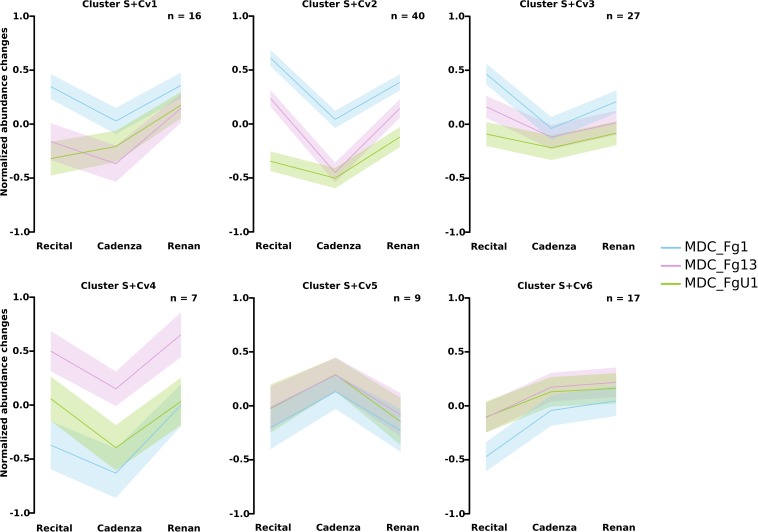
Clustering of *F. graminearum* Strain+Cultivar_effect protein abundance patterns. Clustering was computed using the fuzzy C-means methods using *Z*-score transformed values to identify homogeneous patterns. For each cluster, the average profile was represented by the line plot with the wheat cultivar in *x*-axis and a color code for the *F. graminearum* strains (blue: MDC_Fg1; pink: MDC_Fg13, and green: MDC_FgU1). Ribbons indicate the confidence interval at 5% following the same color code than the line plot. The number of proteins included in each cluster are specified in the right upper corner (n).

Two-way ANOVAs computed to evaluate the significant differences in protein abundance between strains allowed the identification of 255 proteins (i.e., *Strain_effect* proteins and *Strain*+*Cultivar_effect* proteins; Figure 6). Among these proteins, 24 matched with predicted secretion features, including 15 proteins harboring a plastid transit peptide (Supplementary Table S2). In addition, 35 *F. graminearum* proteins were predicted to be effectors according to EffectorP2.0 among which three were also predicted to target the wheat chloroplast or vacuole. A fuzzy C-means clustering of the whole set of these proteins evidenced five consistent clusters (S1–S5; Figure 7A) including 8–49 proteins for *Strain_effect* proteins and six clusters including 7–40 proteins for the *Strain*+*Cultivar_effect* proteins (S+Cv1 to S+Cv6; Figure 8). Six clusters (i.e., Clusters S1, S2, S5, S+Cv1, S+Cv2 and S+Cv3) containing a total of 187 proteins (i.e., nearly 65%) showed abundance that was significantly higher in the strain MDC_Fg1 than in the two other strains. Clusters S2 and S+Cv2 appeared to be enriched in proteins involved in “translation,” “ribosome” and “Protein synthesis” functions and Cluster S5 in proteins involved in “ATP binding” and “carbohydrate metabolic process” functions. These three clusters were also enriched in predicted effector proteins. In all other clusters, fungal protein abundances were lower in MDC_Fg1 than in the two others strains. Cluster S+Cv4 included proteins systematically of higher abundance for MDC_Fg13 while in the cluster S3 no significant difference was found between MDC_Fg13 and MDC_FgU1 protein abundances. No significant protein function enrichment was found in the cluster S+Cv4 while enrichments in “GTP binding,” “GTPase activity,” “ATP binding” and “protein binding” were found in cluster S3. Cluster S4 contained eight proteins whose abundance was the highest in MDC_FgU1, intermediate in MDC_Fg1 and the lowest in MDC_Fg13. No significant enrichment was found in this cluster.

### Does the Host Cultivar Modulate Fungal Protein Abundance Profiles?

Overall, in this experiment the wheat cultivar had a lower effect on the observed variances than the “Strain” factor. For symptoms severity, the percentage of the variance explained by the “Cultivar” factor was not significant regardless of the time point, while it was between 55 and 83% for the “Strain” factor (Supplementary Table S1). The same observation was made for fungal mass monitoring, when the strain genetic background used for infection accounted for 31 and 66.5% of the observed variance at 72 and 168 hpi, respectively, no significant differences between the three wheat cultivars were found with 1.4 and 5.8% of the explained variance (Supplementary Table S1). Concerning DON synthesis rate, significant effects of the “Cultivar” factor were found with nearly 19.5 and 22% of the explained variance at 72 and 168 hpi, respectively (Supplementary Table S1). For these three variables, no specific interaction where found between *F. graminearum* strains and wheat cultivars (Supplementary Table S1).

At the proteomic scale, all proteins whose abundance variations were explained at least in part by the Cv were considered here. This allowed for the identification of 199 fungal proteins including 82 proteins whose abundance changes were explained only by the wheat cultivar (*Cultivar_effect* proteins) and 117 whose abundance changes were explained both by strain and cultivar genetic backgrounds (*Strain*+*Cultivar_effect* proteins, Figure 6). Two proteins showed an effect of the interaction (*Strain*×*Cultivar* proteins). Clustering of these proteins allowed the construction of four consistent clusters (Cv1 to Cv4; Figure 7B) including from 14 to 24 proteins for *Cultivar_effect* proteins and the six *Strain*+*Cultivar_effect* proteins clusters already described (S+Cv1 to S+Cv6; Figure 8). Analysis of the abundance changes revealed that clusters Cv2 and S+Cv6 grouped *F. graminearum* proteins whose abundances were maximal on the low susceptible cultivar Renan and minimal in the highly susceptible cultivar Recital. Clusters Cv1 and S+Cv5 contained *F. graminearum* proteins whose abundance was higher in the cultivar Cadenza than the two others. In contrast, all other clusters (i.e., Cv3, Cv4, S+Cv1, S+Cv2, S+Cv3, and S+Cv4) were characterized by proteins displaying weaker abundances in Cadenza samples than in the two others. Indeed, a total of 20 candidate effectors and 21 putative secreted proteins including 14 proteins harboring a plastid transit peptide were found in these protein clusters for which no significant enrichment was detected.

## Discussion

During this work, we evaluated the aggressiveness of three strains of *F. graminearum* by analyzing three variables conventionally used to explain the disease severity (i.e., symptom severity, DON synthesis rate, and fungal mass) and by investigating their proteomes. For the three variables, significant differences between *F. graminearum* strains were found. This result was mostly discriminating for symptom severity monitoring which allowed for the establishment of a clear ranking of our strains on the three wheat cultivars. However, neither the strain’s level of DON synthesis rate nor the fungal mass quantified *in planta* seems to explain the differences in symptom severity. For example, DON synthesis rate measured in the Recital samples inoculated with MDC_Fg13 was significantly higher than that measured in MDC_FgU1 samples while the symptom severity was not different at the same time. Several studies had already made the same observation (Bai et al., 2001; Birzele et al., 2002; Gilbert et al., 2002; Champeil et al., 2004; Alvarez et al., 2010; Hallen-Adams et al., 2011; He et al., 2019). We can also wonder about the links between the DON synthesis rate and symptom severity with the fungal mass present in the wheat spike. Previous studies showed a strong correlation between fungal biomass and DON production (Lamper et al., 2000; Bai et al., 2001) and between fungal development and symptom severity (Burlakoti et al., 2007) while other studies have not shown any obvious relationship between these parameters (Nicholson et al., 1998; Paul et al., 2005; Alvarez et al., 2010; Hallen-Adams et al., 2011). In this work, no clear correlation between fungal mass, DON synthesis rate and symptom severity was found. The same fungal mass quantified in the three strains at 72 hpi produced different DON amount and induced different symptom severity in the three wheat cultivars. Moreover, in agreement with a previous study showing that the *F. graminearum* genes involved in DON synthesis were highly expressed in the early stages of fungus development (Hallen-Adams et al., 2011), our results suggest that in the most aggressive strain MDC_Fg1, a small amount of mycelium at 72 hpi produced a large amount of DON while a larger quantity of mycelium (168 hpi) resulted in a lower production. Individually, these parameters allow us to efficiently distinguish the different strains but they do not seem to be sufficient to explain their respective aggressiveness.

### *Fusarium graminearum* Strains Express the Same Genetic Program During Wheat Infection

The study of proteins, molecules directly responsible for cell activity, is essential for understanding plant-pathogen interactions (Quirino et al., 2010) and particularly the proteinous effectors that are known to be closely related to the infection success. They are defined as molecules that modify host cell structure and function, allowing the pathogen penetration into host tissues, inhibiting the plant immune responses and manipulating plant physiology for the pathogen’s benefit (Stergiopoulos and de Wit, 2009; Giraldo and Valent, 2013; Selin et al., 2016; Toruño et al., 2016). Comparison of proteins identified in this experiment with those identified in our previous work conducted with the most aggressive strain MDC_Fg1 (Fabre et al., 2019) showed that 85% of the total proteins including 80% of the effectors are common to both experiments. These results highlight the high reproducibility of this analytical method and suggest that there may be a core proteome expressed during the interaction with wheat. This assumption is supported by the fact that nearly 100% of the identified proteins were found in the theoretical proteomes of the three *F. graminearum* strains including all the predicted *F. graminearum* effectors. A similar observation was already made during the analysis of seven strain proteomes of *Mycobacterium tuberculosis* (Peters et al., 2016). This is an interesting result because it suggests that the three *F. graminearum* strains use the same protein set during the early stage of wheat infection and that differences in aggressiveness observed are probably not due to strain-specific proteins but could rather be due to differences in proteins accumulation.

### *Fusarium graminearum* Aggressiveness Could Be Linked to Protein Synthesis and Accumulation During Wheat Infection

Among all the fungal proteins quantified, nearly 76% showed significant abundance differences between the three *F. graminearum* strains. Fungal mass quantification by qPCR at this stage of infection did not identify any differences between the three strains implying that the variations in protein abundance observed are not due to differences in development but rather to a consequence of differential gene expression or protein synthesis. This hypothesis is supported by the significant enrichment in proteins involved in “Protein biosynthesis,” “Ribosome” and “translation” found in the clusters containing proteins whose abundance is maximal for MDC_Fg1. This suggests that the most aggressive strain could have a more efficient protein biosynthesis machinery than the other two strains, which could have a significant effect on the effector abundance produced by this strain. Observation of the predicted effector distribution in the different abundance pattern clusters supports this hypothesis since 67% of them were located in clusters whose abundance is maximal in the MDC_Fg1 strain, intermediate or weak in the MDC_Fg13 and always weak in the MDC_FgU1 strain (Supplementary Table S2). In order to confirm the impact of protein abundance variations on the *F. graminearum* strains aggressiveness, an additional search for proteins already described in the literature was carried out. A total of 16 proteins showing significant abundance changes were matched with proteins encoded by genes known to have a role in *F. graminearum* aggressiveness toward bread wheat (Supplementary Table S2). Among them, all proteins whose abundance was significantly different between the three strains showed higher abundance in samples infected with the most aggressive strain MDC_Fg1 than with the two others strains (i.e., Clusters S1, S2, S+Cv2, and S+Cv3) with the exception of two proteins (FG001_12286 and FG001_06046; Cluster_S5) for which no difference in abundance was observed between MDC_Fg1 and MDC_Fg13. For example, FGSG_07329 included in cluster S2 is a glycogen synthase kinase involved in the activation of many stress-related genes. Mutation of this gene is known to cause a total loss of virulence in *F. graminearum* as well as many pleiotropic effects such as the inability to produce DON (Qin et al., 2015). The presence of this protein in the cluster S2 can be related to the DON synthesis rate results which showed that MDC_Fg1 was the one that globally had the highest production at 72 hpi. This is consistent with the presence of the protein FGSG_07896 in the cluster S5 known to be a Trichothecene 3-O-acetyltransferase involved in the DON self-protection mechanism in *F. graminearum* (Kimura et al., 1998; McCormick et al., 1999). These results agree with those obtained for the analysis of the effector distribution within the different clusters and show a strong correlation with the symptom severity and linked with the DON synthesis rate observed *in planta*. The differences in aggressiveness might not be explainable by strain-specific proteins, but rather by abundance differences in common proteins and effectors having a quantitative effect on the *F. graminearum* pathogenicity at the same development stage.

### Host Cultivar Drives Part of the Fungal Protein Regulations

While many studies have demonstrated that wheat cultivars could strongly differ in their response to FHB (Mesterházy et al., 1999; Mesterházy, 2002; Bai and Shaner, 2004), our results indicate that the fungal genetic background is the main factor explaining the differences in aggressiveness during the early disease development. At the molecular level, the additional power provided by proteomics has made it possible to identify a Cv on the abundance variations of 199 proteins (i.e., 82 *Cultivar_effect* proteins and 117 *Strain*+*Cultivar_effect* proteins). Concerning the *Strain*+*Cultivar_effect* proteins, the *F. graminearum* protein abundance level was mainly determined by the genetic background of *F. graminearum* while differences were amplified when facing different host cultivars. The analysis of the diversity of the *Cultivar_effect* proteins did not reveal significant effector enrichment in the different clusters but five proteins known to be important for *F. graminearum* virulence were found in clusters Cv2 and Cv4. Among these five proteins, we found an arabinanase (FGSG_03598) known to enhance wheat susceptibility by suppressing plant immunity (Hao et al., 2019) and FgTRI14 protein (FGSG_03543) involved in DON synthesis (Dyer et al., 2005). Furthermore, no specific Strain×Cultivar interactions (called *Strain*×*Cultivar_effect* in this work) were found neither in symptom severity, DON synthesis rate and fungal mass monitoring, nor at the proteomics level where only two *Strain*×*Cultivar_effect proteins* were detected. Although the use of only three wheat cultivars cannot represent the whole genetic variability for FHB susceptibility, this illustrates the pivotal role of the fungal component in the disease. *F. graminearum* is a broad-host pathogen (Harris et al., 2016) and it is known that pathogens able to infect multiple hosts have a reduced selection pressure to co-evolve with a particular host, especially when the hosts belong to the same species (Woolhouse et al., 2002). Previous work done on the interaction between a generalist pathogen and cereals has shown that Strain×Cultivar interactions could have only a minor effect, for example in *Puccinia coronata* f. sp. *avenae/Avena sativa* interaction (Bruns et al., 2012), in *Septoria tritici*/*Triticum aestivum* interaction (van Ginkel and Scharen, 1988) or in *Fusarium graminearum*/*Triticum aestivum* interaction (Bai and Shaner, 1996; Xue et al., 2004; Mesterházy et al., 2005). All together, these results indicate that the host cultivars similarly influence the three *F. graminearum* proteomes and that strain-specific protein accumulations did not depend on the host cultivar.

## Conclusion

This study expands our understanding of the determinants of *F. graminearum* aggressiveness during the early stage of bread wheat infection. Three *F. graminearum* strains express the same proteome *in planta* while differences were only detectable in protein abundance. Host background effect was only shown at the protein abundance level and in the DON synthesis rate without any change in the strain ranking. In addition, the absence of Strain×Cultivar specific interaction suggests a common infection strategy of the three strains and modulated identically by wheat cultivars of contrasted FHB susceptibility. Now, confirming these results using more strains and cultivars on a longer infection dynamics will be an additional work in order to understand the determinism of pathogen’s aggressiveness during the interaction.

## Data Availability Statement

The mass spectrometry proteomics data have been deposited to the ProteomeXchange Consortium via the PRIDE (Vizcaíno et al., 2016) partner repository with the dataset identifier PXD015139.

## Author Contributions

TL and LB designed the research. FF and SR prepared the samples. FF, SU, and LB performed the proteomic experimentation. JB involved in deoxynivalenol synthesis rate measurements. FF and JB involved in qPCR measurements. FF and LB analyzed the data, prepared the figures, and wrote the manuscript. LB conceived and performed modeling.

## Conflict of Interest

The authors declare that the research was conducted in the absence of any commercial or financial relationships that could be construed as a potential conflict of interest.

## Footnotes

1 http://www.thegpm.org/TANDEM/

## Abbreviations

ANOVA: Analysis of variance
Cv: Cultivar effect
DON: Deoxynivalenol
FDR: False discovery rate
FHB: Fusarium head blight
hpi: hours post-inoculation
*S*+*Cv*: *Strain*+*Cultivar effect*
*S* × *Cv*: *Strain* × *Cultivar effect*
*S*: *Strain_effect*.
